# Incorporating health care quality into health antitrust law

**DOI:** 10.1186/1472-6963-8-89

**Published:** 2008-04-22

**Authors:** Helen Schneider

**Affiliations:** 1 Nicholas C. Petris Center on Health Care Markets & Consumer Welfare, University of California at Berkeley, Berkeley, CA 94720, USA; 2 University of Texas at Austin, Austin, TX 78712, USA

## Abstract

**Background:**

Antitrust authorities treat price as a proxy for hospital quality since health care quality is difficult to observe. As the ability to measure quality improved, more research became necessary to investigate the relationship between hospital market power and patient outcomes. This paper examines the impact of hospital competition on the quality of care as measured by the risk-adjusted mortality rates with the hospital as the unit of analysis. The study separately examines the effect of competition on non-profit hospitals.

**Methods:**

We use California Office of Statewide Health Planning and Development (OSHPD) data from 1997 through 2002. Empirical model is a cross-sectional study of 373 hospitals. Regression analysis is used to estimate the relationship between Coronary Artery Bypass Graft (CABG) risk-adjusted mortality rates and hospital competition.

**Results:**

Regression results show lower risk-adjusted mortality rates in the presence of a more competitive environment. This result holds for all alternative hospital market definitions. Non-profit hospitals do not have better patient outcomes than investor-owned hospitals. However, they tend to provide better quality in less competitive environments. CABG volume did not have a significant effect on patient outcomes.

**Conclusion:**

Quality should be incorporated into the antitrust analysis. When mergers lead to higher prices and lower quality, thus lower social welfare, the antitrust challenge of hospital mergers is warranted. The impact of lower hospital competition on quality of care delivered by non-profit hospitals is ambiguous.

## Background

Economic theory suggests that competition leads to efficient outcomes. The health industry, however, is dominated by non-profits that have different objectives than the for-profit agents. Therefore, in health care markets, the impact of competition on pricing and social welfare in general is uncertain. To improve social welfare, antitrust litigation ensuring that health care markets are competitive should result not only in lower prices and costs but in higher health care quality as well. Very little evidence is currently available on the correlation between hospital pricing and quality and the effect that hospital competition has on the many dimensions of health care quality. While numerous studies examine the impact of hospital competition on hospital prices and costs, the impact of competition on hospital quality has received little attention and the available empirical evidence is ambiguous [1-4].

Antitrust authorities and policy-makers usually treat price as a proxy for quality and consumer welfare, in part because health care quality is difficult to observe. Recently, however, the ability to measure quality has improved greatly. Hence, it is becoming more widely available in an effort to help consumers and purchasers make the informed choices. From beta-blocker utilization to risk-adjusted mortality rates researchers are using a wide variety of measures to assess and rank health care providers [5]. Therefore, the examination of the relationship between competition and quality of health care services has become an important research topic as well as a public policy issue [6]. Cuellar and Gertler (2003) suggest that higher post-merger hospital prices may reflect higher quality [7]. However, this claim is not supported by empirical evidence. Although mergers do lead to higher prices and expenditures there is mounting empirical evidence that expanded hospital competition does not improve all dimensions of hospital quality [3,4,8]. Shortell and Hughes (1988), for example, find no significant association between hospital competition and inpatient mortality rates [9]. This result may be due to the time period the study was conducted. Kessler and McClellan (2000) found that competition significantly reduces costs as well as adverse health outcomes, especially for the time period after 1990 [10]. Before 1991 the study found that competition led to higher costs and higher quality of care. Ho and Hamilton (2000) compared the quality of hospital care before and after mergers in California between 1992 and 1995 [11]. They found no evidence that mergers and acquisitions measurably affected inpatient mortality. However, they did find that hospital consolidations are associated with increased readmission rates for heart attack patients in some cases and increased likelihood of early discharge for normal newborns. Mukamel et al. (2001) found no significant association between hospital competition and risk-adjusted hospital mortality rates [12]. Sari (2002) showed that both higher hospital market share and market concentration are associated with lower quality of care as measured by the in-hospital complications [13]. Similarly, Rivers and Fottler (2004) found that more competitive markets have lower risk-adjusted mortality rates [14]. Therefore, previous empirical findings indicate that quality should be incorporated into antitrust analysis. If mergers are indeed shown to lead to higher prices and lower quality (or no quality improvements at all), thus lowering social welfare taken as a whole, the case to challenge hospital mergers is significantly strengthened [13].

### Importance of the study

This study examines the relationship between hospital competition and hospital quality along one dimension: risk adjusted mortality rates following Coronary Artery Bypass Graft (CABG). Analyses of how hospital competition affects quality measures for heart disease are of special importance since one-sixth of total hospital expenditures are devoted to treatment of heart disease and because such analyses may generalize to other acute illnesses [10,15]. In California, over 120 non-federal hospitals offer bypass surgery to about 27,000 adult patients each year over the time period studied, 1997–2002 [16]. In addition, the impact of competition on CABG surgery is of special interest because CABG is rarely performed in emergency settings. Thus, hospitals may be relatively more interested in improving CABG care in response to competition as opposed t procedures that require immediate admission and allow for little hospital choice such Acute Mycardial Infraction (AMI) care. However, there is also an opportunity for hospital selection by patients although the impact of public reporting of risk-adjusted mortality rates on hospitals' market share is uncertain [17,18]. Such non-random hospital selection may pose econometric problems and lead to biased coefficients.

CABG is one of the most frequently performed and costly surgeries. It is also considered a very safe surgery and is associated with quite low death rates (about 2.8% of all patients) [16]. However, the total number of CABG surgeries performed has been declining over time as balloon angioplasty and drug-coated stents have replaced it in many less complicated cases. Although mortality data in California is in line with national levels, the majority of California hospitals perform fewer bypass surgeries each year than is recommended for best outcomes by the American College of Cardiology and American Heart Association [19]. In contrast, New York hospitals perform a higher volume of surgeries and report lower mortality rates. Thus, the impact of hospital volume on quality is of special policy interest in states like California. Although some previous studies suggest an inverse relationship between hospital volume and mortality [20,21], this result has been challenged in more recent work [22-24].

### Contribution of this study to previous research

This study contributes to the existing body of literature along several dimensions. First, we focus on risk-adjusted mortality rates following CABG surgery, a procedure that has not been closely examined. Second, we use alternative measures of hospital market to measure the extent of hospital competition. Third, this study closely examines the quality outcomes of non-profit hospitals in some more monopolistic markets, a topic that caused much controversy among researchers. Lastly, the study attempts to examine the relationship between hospital-specific CABG mortality rates and CABG volume.

## Methods

### Data sources

The study includes 373 hospitals that performed at least two CABG surgeries over the period 1997–2002. Hospital data have been obtained from the Hospital Annual Financial Disclosure Reports filed annually by all California hospitals with the Office of Statewide Health Planning and Development (OSHPD). Risk adjusted mortality rates are based on the California Coronary Artery Bypass Graft (CABG) Mortality Reporting Program (CCMRP). The hospital-level data represents more than 70% of all CABG surgeries performed in California. Data submitted by each hospital was reviewed for completeness and errors by OSHPD via an independent medical records audit performed for some years on selected hospitals.

Managed care enrollment estimates were based on managed care enrollment data provided by Cattaneo and Stroud Inc. study funded by the California Health Care Foundation. Managed care customers in managed care commercial, Medi-Care, Medi-Cal and Healthy Families programs.

The source for the year- and county-specific income per capita was the U.S. Department of Commerce, Bureau of Economic Analysis.

### Empirical model

In this study we used the risk-adjusted mortality measures developed by OSHPD. Risk-adjusted mortality rates have been widely used as indicators of health care quality since they capture systematic differences in quality across hospitals and account for patient risk factors that are beyond a hospital's control. Failing to adjust for patient risk factors may give rise to omitted variable bias and result in incorrect results concerning the relationship between hospital competition and hospital quality. The unobservable severity of illness is especially important for CABG where hospital choice is not always limited by the urgency of care. A logistic regression risk model was used to adjust for patient characteristics: age, gender, body mass index, acuity (elective, urgent, emergent or salvage) and secondary conditions (such as hypertension, diabetes, etc.). Excess mortality is defined as the difference between the observed mortality rate and the predicted rate.

Let q_ht _denote the quality measure, excess mortality, for hospital *h *in year *t*. Our empirical model can be written as:

q_ht _= αMarket_ht _+ βInstitutional_ht _+ δ(Ownership*HHI) + γEnvironmental_ht _+ ζ(Public Insurance*HHI) + λTime + μ_h _+ ε_ht_,

where the market characteristics include both the hospital competition index and managed care penetration. Hospital competition was measured by the Herfindahl-Hirschman Index (HHI), defined as the sum of squared market shares of all hospitals competing in the same market. We define a hospital market as the health service area (HSA) specified by the National Center for Health Statistics [15,25]. This definition uses an algorithm that minimizes the travel distance to hospitals. Thus, unlike counties or Metropolitan Statistical Areas (MSAs), HSAs rely on patient flows rather than geo-political boundaries to define hospital markets. There are 14 HSAs in California. The HHI index was constructed for each HSA and year. Since hospital competition measures based on actual flows of patients may be endogenous, in our sensitivity analysis we use an alternative definition based on county geographic boundaries. There are two important market definitions in hospital merger cases, geographic market and product market (CABG market in our case). First, we estimate HHI for all hospitals in the market based on staffed beds. Such measure will capture general hospital competition that may spillover into the CABG market. Additional analyses were conducted in which we defined the market share based only on admissions for CABG surgery. Thus we present results separately for the hospital and CABG markets.

Besides hospital competition, market characteristics in (1) include HMO (health maintenance organization) penetration. It is defined as a number of people enrolled in managed care divided by the total population. The data was obtained on HMO penetration for each county and year in the sample.

The vector of institutional characteristics in Equation (1) includes hospital ownership types, system affiliation, teaching status, and hospital size (number of staffed beds). Ownership indicator variables included non-profit, district and county ownership. The omitted category was investor-owned ownership. Since non-profits may respond to changes in competitive environment differently than for-profits [2], we include interaction variables between hospital ownership and competition measure. Teaching hospitals were defined as hospitals with some residents. The institutional variables also contain interactions between ownership status and HHI, since non-profits can respond differently than for-profits to competitive pressures. Given that changes in the outcomes may represent a volume effect, the number of patients admitted for CABG surgery in the hospital during the year is also included.

Environmental characteristics in (1) include per capita personal income and percent of Medicare and Medi-Cal (California Medicaid program) patients. These variables capture patients' ability to pay for medical care and health care coverage across hospitals. Public insurance data is hospital-specific and is reported by OSHPD. Gaynor and Vogt (2003) suggest that competition should have a higher effect on quality when the prices are fixed – for example for Medicare and Medi-Cal patients – than when the prices are variable [26]. Thus, as hospitals gain market power (as their HHI goes up), the quality of care may fall due to competition for patients whose payers pay fixed, non-negotiable fees. We test this hypothesis by adding interaction variables between HHI and hospital Medicare and Medi-Cal penetration variables.

The vector of time variables in (1) includes year dummies to control for the time trend. Finally, we include hospital-specific fixed effects (μ_h_) to account for time-invariant differences. Since fixed effects capture all characteristics that do not change over the sample period (e.g. county ownership), we present our results both with and without the fixed effects.

## Results

### Descriptive summary

Table 1 presents descriptive statistics of market characteristics for each of the 14 HSAs. Also, we include mean CABG volume to better illustrate the size of each market. The average number of hospitals performing CABG in each market varies from 1 hospital to 19 per year. Therefore, hospital competition varied significantly from very competitive HHI of 0.095 to monopoly in the CABG market (i.e. HHI of 1). Although each HSA includes several contiguous counties, there is a lot of variation across markets. For example, mean HMO penetration varies from 12.9% to over 70%. Similarly, mean CABG volume varies from 134.4 to 410.53 procedures.

**Table 1:** Health Service Area (HSA) characteristics, 1997–2002

HSA	Mean hospitals per year	Minimum hospitals per year	Maximum hospitals per year	Mean market HHI (st. deviation)	Mean CABG HHI (st. deviation)	Mean CABG volume (st. deviation)	Mean HMO penetration (st. deviation)
1	1.83	1	2	0.0507 (0.00489)	0.645 (0.134)	387.91 (217.86)	0.129 (0.0330)
2	3.17	2	4	0.084 (0.00128)	0.545 (0.0907)	410.53 (381.62)	0.705 (0.0116)
3	1.67	1	2	0.114 (0.00843)	0.610 (0.206)	203.6 (47.77)	0.592 (0.0490)
4	6.83	5	8	0.0888 (0.00519)	0.278 (0.0652)	273.98 (279.93)	0.558 (0.0473)
5	4.67	4	6	0.0704 (0.00612)	0.316 (0.0752)	187.61 (169.61)	0.649 (0.0185)
6	3.17	3	4	0.0777 (0.00458)	0.379 (0.0509)	278.52 (130.44)	0.530 (0.0537)
7	1.67	1	3	0.124 (0.0258)	0.675 (0.252)	134.4 (95.08)	0.591 (0.0205)
8	1.83	1	2	0.123 (0.0157)	0.601 (0.136)	197.36 (89.0)	0.271 (0.0127)
9	1.0	1	1	0.0643 (0.0107)	1 (0)	340.33 (52.92)	0.230 (0.0759)
10	2.0	1	3	0.0855 (0.00475)	0.536 (0.227)	196.83 (77.93)	0.419 (0.0211)
11	19.17	17	25	0.0155 (0.00107)	0.095 (0.0305)	208.06 (206.86)	0.485 (0.0403)
12	2.0	1	3	0.0498 (0.00273)	0.386 (0.0797)	282.33 (178.66)	0.540 (0.0328)
13	5.83	1	9	0.0434 (0.00655)	0.187 (0.144)	204.37 (67.38)	0.532 (0.0532)
14	6.33	4	8	0.0551 (0.00179)	0.178 (0.0413)	277.68 (84.74)	0.514 (0.0309)

Table 2 displays mean values, standard deviations and minimum and maximum values for all non-interaction variables included in the analysis. The mean market HHI – around 0.0554 – suggests that hospital markets are very competitive (markets with HHI below 0.18 are considered moderately or very competitive) [27]. However, the mean HHI based on CABG volume is 0.29, with a mean range between 0.095 and 1 for some markets. This suggests that there is large variation in HHI across CABG markets. Many hospitals were located in a competitive environment, with 42.6% of hospitals in markets with a HHI below 0.18.

**Table 2:** Descriptive statistics for selected variables, 1997–2002 (N = 373)

Variable	Mean (st. deviation)	Minimum	Maximum
*Outcome measures*			
Observed Mortality	3.15 (1.60)	0	12.1
Expected Mortality	2.93 (1.03)	0	6.89
Excess mortality	0.213 (1.37)	-3.63	6.84

*Market characteristics*			
Market HHI	0.0554 (0.185)	0.0143	0.169
CABG HHI	0.290 (0.225)	0.057	1
HMO penetration	0.512 (0.116)	0.043	0.724

*Ownership status*			
County	0.535% (07.30)		
District	7.23% (25.9)		
Non-profit	81.3% (39.1)		
Investor-owned	11.0% (31.3)		

*Hospital characteristics*			
Teaching	16.9% (37.5)		
System affiliation	56.0% (49.7)		
CABG volume	236.64 (197.40)	15	1531
Staffed beds	315.1 (153.63)	0	875
Per capita income	32.53 (9.21)	17.51	69.35
% Medicare	30.20 (16.76)	0	84.86
% Medi-Cal	12.71 (10.69)	0	59.31

*Time variables*			
1997	13.7% (34.4)		
1998	19.8% (39.9)		
1999	17.4% (38.0)		
2000	16.6% (37.3)		
2001	16.6% (37.3)		
2002	15.8% (36.5)		

Excess mortality averaged above zero at 0.213 with 49.6% of hospitals performing above zero, i.e., having observed mortality above expected.

### Regression results

Table 3 and Table 4 present multivariate regression results. The results indicate that hospital competition negatively affects excess mortality. Thus, as markets become more monopolized (as HHI approaches 1) quality of care as measured by a risk-adjusted mortality decreases. This result holds for both measures of hospital market, with and without hospital-specific fixed effects. Marginal effects of HHI not reported in Table 3 are 25.32 and 2.87 for the two market definitions; both effects are statistically significant. Non-profit hospitals are no different in quality than the investor-owned hospitals. However, in more concentrated markets non-profit hospitals tend to provide higher quality of care relative to for-profits.

**Table 3:** Excess mortality-multivariate regression results, 1997–2002

Variable	Hospital Market	CABG Market
	
	Estimate (st. error)	Estimate (st. error)
HHI	26.04*** (9.01)	3.07** (1.22)
HMO penetration	0.578 (0.706)	0.958 (0.752)
Non-profit	0.554 (0.477)	0.265 (0.373)
County	-0.757 (1.11)	-0.813 (1.07)
District	-0.120 (0.888)	-0.340 (0.582)
System	0.116 (0.156)	0.062 (0.152)
Teaching	0.409* (0.247)	0.390 (0.242)
Staffed beds	-0.00104* (0.000617)	-0.00107* (0.000607)
CABG Volume	-0.00133*** (0.000354)	-0.00146*** (0.000357)
Per capita income	0.0130 (0.00959)	0.00855 (0.00879)
% Medicare	-0.0207** (0.00929)	-0.0220** (0.00898)
% Medi-Cal	0.0310** (0.0121)	0.0267** (0.0107)
Non-profit*HHI	-68.98*** (19.46)	-3.78*** (0.973)
District*HHI	-25.55** (12.59)	-2.58** (1.24)
% Medicare*HHI	0.0916 (0.132)	0.0113 (0.0222)
% Medi-Cal*HHI	-0.539** (0.250)	-0.0702* (0.0385)
1997	0.107 (0.267)	-0.00284 (0.263)
1998	-0.113 (0.243)	-0.219 (0.241)
1999	-0.200 (0.247)	-0.248 (0.245)
2000	0.0874 (0.237)	0.0527 (0.237)
2001	-0.510 (0.395)	-0.601 (0.393)
Constant	0.0750 (0.806)	0.874 (0.716)
R-squared F	0.180 3.64***	0.189 3.88***

Note: *P < 0.10, **P < 0.05, ***P < 0.01, Heteroskedasticity corrected standard errors are in parentheses.

**Table 4:** Excess mortality-fixed effects regression results, 1997–2002

Variable	Hospital Market	CABG Market
	
	Estimate (st. error)	Estimate (st. error)
HHI	31.53** (15.51)	5.00*** (1.77)
HMO penetration	-3.00 (2.04)	-0.981 (2.27)
Non-profit	-5.20* (2.65)	0.418 (0.851)
District	-5.60* (2.95)	2.48 (1.53)
System	-0.185 (0.309)	-0.0167 (0.303)
Teaching	3.66*** (1.15)	0.244 (0.258)
Staffed beds	-0.000849 (0.00161)	-0.000609 (0.00154)
CABG Volume	-0.000399 (0.00091)	-0.0000602 (0.000900)
Income per capita	0.0133 (0.0251)	-0.0292 (0.0262)
% Medicare	-0.0137 (0.0116)	-0.0232** (0.00943)
% Medi-Cal	0.00470 (0.0204)	0.0299*** (0.0111)
Non-profit*HHI	-69.86*** (21.01)	-4.33*** (1.04)
District*HHI	-35.13** (17.34)	-8.18*** (2.90)
% Medicare*HHI	0.139 (0.138)	0.0125 (0.0249)
% Medi-Cal*HHI	-0.568** (0.283)	-0.0786* (0.0438)
1997	0.287 (0.311)	-0.202 (0.280)
1998	0.125 (0.347)	-0.322 (0.249)
1999	-0.00694 (0.231)	-0.351 (0.253)
2000	0.0846 (0.189	0.030 (0.235)
2001	-0.466 (0.462)	-0.521 (0.414)
Constant	3.80 (2.70)	3.10** (1.42)
R-squared F	0.127 2.96***	0.127 3.19***

Note: *P < 0.10, **P < 0.05, ***P < 0.01, Heteroskedasticity corrected standard errors are in parentheses.

Estimates of the effects of Medicare and Medi-Cal case loads yielded interesting results. The presence of higher Medi-Cal case load in a given hospital leads on average to a significantly lower quality. In more concentrated markets, however, such hospitals are seen to provide higher quality. Thus, safety-net hospitals with a large volume of Medi-Cal patients may not cut on quality in the presence of low competition relative to other health care providers. Hospitals with a higher Medicare load have, on average, higher quality. However, in the more concentrated markets such hospitals tend to have worse outcomes, as hypothesized by Gaynor and Vogt (2003) but the estimates are not statistically significant after we adjusted for fixed effects [26]. Alternative explanations of these results include profit margins for CABG for Medicare and Medi-Cal patients. More research is necessary to examine the effect of Medicare and Medicaid penetration on hospital risk-adjusted mortality rates and other measures of quality.

The multivariate regression results in Tables 2 and 3 show that hospitals that perform a higher volume of CABG procedures report significantly better outcomes. However, once we add hospital-specific fixed effects the significance of volume disappears. In general, regression results of the effect of volume on clinical outcomes should be interpreted with caution due to potential endogeneity. The presence of unobservable factors and potential reverse causality in CABG markets where patients may search for the health care provider based on quality may bias these estimates. Two stage least squares (TSLS) results that correct for endogeneity show that although the relationship between volume and excess mortality is positive, it is not statistically significant. The key identifying variable was the volume of other surgical procedures performed in the hospital. We assume that the volume of other procedures does not affect CABG mortality but does affect CABG volume. The instrument used (volume of other surgical procedures) is a significant predictor of CABG volume and passed the Staiger-Stock test for weak instruments [28]. Although there is no significant correlation between volume of other surgical procedures and CABG outcomes, the relationship may be indirect through hospital size. Despite controlling for the size of the hospital a better instrument may improve our understanding of the relationship between CABG volume and CABG outcomes. The use of a weak instrument is one of the limitations of this study.

Our TSLS results are consistent with findings by Ho (2000) and Tsai et al. (2006) that show that over time, the disparity in outcomes between low- and high-volume hospitals has narrowed [23,24]. More specifically, Welke et al. (2005) found that hospital volume is not a discriminator of mortality for CABG [29].

Other significant variables include interaction between district ownership and competition; such hospitals have lower excess mortality in more concentrated markets in all specifications of the model. After hospital fixed effects adjustment we find that the association between HMO penetration and quality is negative (areas with higher penetration had lower risk-adjusted mortality rates) but not statistically significant. Thus, managed care does not significantly decrease quality as is often hypothesized.

### Sensitivity analyses

Mukamel, Dick and Spector (2000) show that even under best case assumptions and perfect risk adjustment different measures of risk-adjusted mortality outcomes may lead to incorrect ordering of providers [30]. Our results are robust to alternative specifications of the model. The use of alternative measures of risk-adjusted CABG mortality, for example taking observed to expected mortality ratios above 1.0 and the differences between hospital observed to expected mortality ratios and this ratio for the cohort did not change the main conclusions of this research.

Measures of competition that are based on actual patient flows (HSAs) may be endogenous due to higher quality hospitals attracting more patients from far away areas. Defining hospital market based on geographic boundaries (counties) rather than patient flows (HSAs) did not change our results concerning the impact of competition with respect to excess mortality.

To check for potential omitted variable biases, HSA random effects were added to the model in (1). Random effects model also allows us to generalize our results to markets beyond California. HSA random effects results are consistent with those presented in Tables 3 and 4. The coefficient on hospital market HHI is 22.79 with a p-value of 0.025. In the CABG market the coefficient is smaller in magnitude than what we observe in Tables 3 and 4 (0.0679) with a p-value of 0.093. Despite using California data, our random effects results allow us to generalize conclusions of this study to all markets: hospital competition has a positive and statistically significant effect on patient outcomes.

Since hospital participation in the OSHPD survey did not include all of the hospitals performing CABG (only 70% of the CABG surgeries performed in California were represented) further adjustments were conducted for potential hospital selection into the survey sample. Heckman adjustment of selection into the sample did not change the main conclusions of this research.

## Discussion

### Policy implications

The ability of competition in health care markets to control rising health care costs depends on its impact on health care prices and patient outcomes. Currently the impact of competition in health care markets is subject to controversy due to the presence of non-profits in the industry. The perception that non-profit hospitals are different from for-profits in their post-merger behavior is based on Lynk (1995) [2]. In recent antitrust cases non-profit hospitals often argue their exceptionality, citing their non-profit mission and higher post-merger quality, thus effectively preventing the Federal Trade Commission (FTC) from averting what it deems to be anticompetitive mergers [31,32]. There are many theoretical models that explain non-profit behavior, from physician's cooperative at one extreme to identity with consumer cooperatives at the other. Therefore, theoretically, price and quality behavior of hospitals is ambiguous. Recent empirical reassessments of Lynk's analysis show that a reduction in competition leads to an increase in prices regardless of ownership status [3,4,33]. Although the empirical evidence points to higher post-merger prices and costs, the full impact of mergers on social welfare cannot be fully understood enough without considering their impact on quality. This study finds that competition has a significantly positive effect on patient outcomes and leads to lower risk-adjusted mortality rates. Similar results were found in previous research addressing other measures of quality [11,13,14]. Therefore, successful FTC challenges of hospital mergers can potentially improve health care quality. This study also finds that non-profit hospitals are no different from their investor-owned counterparts regarding the risk-adjusted CABG mortality rates. However, they tend to have better quality in more concentrated markets than the for-profit hospitals. Thus, if mergers of non-profit hospitals lead to higher prices and better quality relative to for-profits, the overall effect of such mergers on social welfare is unclear. Finally, our results suggest that the incentives for hospitals to reduce mortality rates vary according to the method of reimbursement. Therefore, both antitrust and Medicare and Medi-Cal policies play a role in determining hospital quality. Higher competition may benefit patients in aggregate but still harm some subgroups. Increases in hospital market power, as would occur following a merger, can lead to increases or decreases in risk-adjusted hospital mortality and the effect depends on hospital ownership as well as Medicare and Medi-Cal penetration.

### Limitations and avenues for future research

We have examined mortality measures for one specific procedure, CABG surgery, and care must be taken when extrapolating these results to other measures since hospitals performing well in one clinical area do not necessarily perform just as well in others [8,34]. Another limitation of choosing CABG mortality rates is potential patient selection of health provider based on quality. The unmeasured severity in markets where patients have an opportunity to select their medical provider may lead to biased estimates. Our analyses indicate that since OSHPD's public release of hospital quality indicators, high-quality hospitals' market share has not significantly changed. This may be due to the fact that such adjustments take time (i.e. disseminating quality information to consumers may be associated with a time lag) or that consumers do not select health care providers based on quality reports.

Results of this study rely on in-hospital risk-adjusted mortality rates to measure quality. We need to remember that although mortality is a critical outcome, it may not closely reflect other important dimensions of quality such as patient satisfaction, morbidity, or probability of readmissions.

One further limitation bears mentioning. We use a sample of California hospitals rather than a nationally representative sample. Thus, some of the relationships may not be universal to other data, specifically those pertaining to the effects of Medi-Cal insurance coverage.

This study analyzes only quality implications of hospital competition. Adding the hospital-specific CABG costs, charges and charges to costs ratios to the analysis would determine more fully how hospital competition affects social welfare.

## Conclusion

Results of this study provide further evidence that quality should be incorporated into the antitrust analysis. When mergers lead to higher prices and lower quality, thus lower social welfare, the antitrust challenge of hospital mergers is warranted. Further, ownership may play a role in hospitals' response to market power. Non-profit hospitals tend to maintain better health care outcomes in the absence of competitive pressures.

## Competing interests

The author(s) declares that they have no competing interests.

## Authors' contributions

HS conceived of the study, designed the study, conducted empirical analysis, and wrote and revised all of the drafts of the manuscript.

## Pre-publication history

The pre-publication history for this paper can be accessed here:

http://www.biomedcentral.com/1472-6963/8/89/prepub
